# Supplementary material for: Metal Exposure, Smoking, and the Risk of COPD: A Nested Case–Control Study in a Chinese Occupational Population
Source: Int J Environ Res Public Health. 2022 Sep 1;19(17):10896. doi: 10.3390/ijerph191710896 (PMC9518333; doi:10.3390/ijerph191710896)
Supplement: Supplementary file 1 [file ijerph-19-10896-s001.zip › ijerph-1840883-supplementary.pdf]

Table S1. Health risk assessment results of heavy metal exposure in the Jinchang cohort

Service Units Classification	Occupation Category	Hazard Level (C)	Exposure Score (Pre)	Exposure Time (PeE)	Uncertainty (U)	Risk Value (RR)	Risk Level
Services and light industries	Service personnel and workers	1	1	0.5	1	0.5	low
Transport Engineering	Office service personnel	1	2	1	2	4	low
	Technical personnel	1	2	2	2	8	low
	Workers	15	4	2	1	120	high
Mechanical maintenance	Office service personnel	1	2	1	1	2	low
	Technical personnel	1	4	2	2	16	low
	Workers	15	5	2	1	150	high
Metallurgical and environment management	Office service personnel	1	3	1	1	3	low
	Technical personnel	1	5	2	2	20	medium
	Workers	15	6	2	1	180	high
Production of chemical raw materials and products	Office service personnel	1	5	1	1	5	low
	Technical personnel	1	6	2	2	24	medium
	Workers	15	9	2	1	270	very high
Metal rolling processing	Office service personnel	1	2	2	1	4	low
	Technical personnel	1	3	2	2	12	low
	Workers	1	6	6	1	36	medium
mining	Office service personnel	15	3	2	1	90	high
	Technical personnel	15	7	2	2	420	extremely high
	Workers	15	8	6	1	720	extremely high
beneficiation	Office service personnel	1	3	2	1	6	low
	Technical personnel	15	6	2	1	180	high
	Workers	15	7	6	1	630	extremely high
smelting	Office service personnel	1	6	2	2	24	medium
	Technical personnel	1	9	2	2	36	medium
	Workers	15	10	6	1	900	extremely high
